# Disparities in Urine Testing Among Febrile Children in US Emergency Departments

**DOI:** 10.1007/s40615-025-02290-3

**Published:** 2025-02-17

**Authors:** Than S. Kyaw, Carina De La Cueva, Natalia Leva, Debbie Goldberg, Isabel E. Allen, Maya Overland, Sam Washington, Hillary L. Copp

**Affiliations:** 1 Medical Scientist Training Program, University of California, San Francisco, San Francisco, CA USA; 2 Department of Urology, University of California, San Francisco, San Francisco, CA USA; 3 Department of Epidemiology & Biostatistics, University of California, San Francisco, San Francisco, CA USA; 4 Division of Urology, Children’s Hospital of Philadelphia, Philadelphia, PA USA

**Keywords:** Urinary tract infection, Urine testing guidelines, Disparities, Emergency department

## Abstract

**Objective:**

To evaluate the association of race and ethnicity with urine testing for febrile children in the emergency department and to measure trends in urine testing from 2002 to 2021.

**Study Design/Methods:**

We conducted a cross-sectional analysis of children aged 2–24 months with a recorded temperature of ≥ 38 °C who presented to an emergency department in the United States from 2002 to 2021 using the National Hospital Ambulatory Medical Care Survey. We assessed trends in urine testing stratified by sex, race, and ethnicity over two decades and performed univariate and multivariate analyses.

**Results:**

Between 2002 and 2021, there were 31,552,201 estimated emergency department visits by febrile children aged 2–24 months with recorded temperature ≥ 38.0 °C. In 2002–2012, Hispanic females and non-Hispanic Black females had significantly lower frequencies (13–19% vs. 26%; *p* < 0.001) compared to non-Hispanic White females. However, urine testing frequencies significantly increased over the study period for Hispanic and non-Hispanic Black females (coefficients of 1.10 and 0.74, respectively; multivariate linear regression tests for trend, *p* < 0.05). By 2021, there were no racial and ethnic differences in urine testing (*p* > 0.05). There were no differences among males (*p* > 0.05).

**Conclusions:**

Nationally, Hispanic and non-Hispanic Black females had disproportionately lower frequencies of receiving urine tests at the beginning of the study period but had equivalent frequencies to non-Hispanic White females by 2021, suggesting that racial and ethnic disparities have decreased over time. Future research will examine the impact of these trends on disparities in health outcomes.

**Supplementary Information:**

The online version contains supplementary material available at 10.1007/s40615-025-02290-3.

## Introduction

Urinary tract infections (UTIs) are common bacterial infections that, if left untreated, may result in long-term consequences of renal scarring for infants and young children [1]. Accurate diagnosis is therefore crucial to prevent the downstream effects of untreated UTIs. Proper diagnosis in pediatric patients is frequently challenging due to non-specific clinical presentations and difficulty in obtaining uncontaminated urine specimens, which may lead to missed diagnoses [1, 2]. However, overdiagnosis of UTI can lead to overtreatment, unnecessary antibiotics exposure, and expensive imaging [3]. To help standardize the decision to test for UTIs in febrile pediatric patients, the American Academy of Pediatrics (AAP) published guidelines for diagnosis and management of UTIs [4, 5].

One of the risk factors that the AAP incorporated into their algorithm for urine testing in their 2011 guidelines was race [3]. Specifically, White race for girls and non-Black race for boys were deemed high risk for pediatric UTIs [3]. However, using race as a proxy for biological differences in medical settings is deeply problematic. It unintentionally implies that the urinary tracts of socially identified White girls and socially identified Black boys are physiologically unique in the capacity to resist bacterial infections, which is not supported by current evidence [6]. It further reproduces racial disparities, justifies structural racism, and translates to substandard care for people of color [6]. Thus, in an effort to reduce systemic racism in medicine, there was widespread support and a call for the removal of race from the AAP guidelines, warning against the downstream consequences that would result in lower urine testing among Non-White children [5]. Shortly, thereafter, a unanimous vote for the removal of race in the AAP UTI guideline occurred in 2021 [7].

Given that our views and practices around race-based medicine have evolved significantly over the last few decades, as evident by changes in the AAP UTI guidelines, we aim to assess. The association of race and ethnicity with urine testing for febrile children in the emergency department settings and to measure trends in urine testing over time, at the national level in the United States (US). We hypothesized that because of our increasing awareness of the detrimental effects of race-based medicine, the racial and ethnic gaps in urine testing would close over the recent years.

## Materials and Methods

### Study Design

We conducted a cross-sectional study to determine whether race and ethnicity contribute to differences in UTI testing frequencies among febrile children aged 2–24 months old who presented to the emergency departments in the United States between 2002 and 2021, the most recently available data, based on the National Hospital Ambulatory Medical Care Survey (NHAMCS). As this study utilizes a publicly available national dataset, it is exempt from the Institutional Review Board review.

### Data Source and Case Identification

We queried the NHAMCS, which is an annual survey published by the Centers for Disease Control and Prevention (CDC) to collect data on the services provided in a nationally representative sample of hospital emergency departments and ambulatory surgery centers [8, 9]. To obtain a nationally representative sample, NHAMCS utilizes a multistage clustered probability sampling method to select physicians to participate based on their geographical location and specialty [8]. Each provider selected to participate was randomly assigned a 1-week reporting period where predetermined data were collected from every patient visit within the selected provider’s reporting timeframe. National estimates were then generated using a weight that was assigned to each patient visit. NHAMCS, which began collecting data on hospital-based ambulatory surgery centers in 2009 and included freestanding centers from 2010 to 2012, ceased outpatient department data collection in 2018 and focused solely on emergency department visits from 2018 to 2022 [10]. Although the patient data record forms changed over the years, the key variables of interests were always captured over the study period.

Our study population consisted of all sampled emergency department visits between 2002 and 2021 by children aged 2–24 months with a recorded body temperature ≥ 38 °C. As we are interested in fever concerning a UTI, we excluded patients presenting with other potential sources of fever, such as postoperative fever, pregnancy-related, traumatic injuries, and infections unrelated to the urinary tract, such as respiratory tract infections and skin infections. We also excluded patients with complex chronic conditions who rely on dialysis catheters, ostomies, prosthetics, implants, or grafts, all of which pose a higher risk for infections. A complete list of International Classification of Disease 9th and 10th revision codes (ICD-9 and ICD-10 codes) used to define our exclusion criteria is in the Supplementary Table 1.

### Exposure of Interest

Our primary exposures of interest were race and ethnicity. NHAMCS utilized information from medical records and surveyor-recorded data, without distinction, to capture race and ethnicity. Race and sex were imputed for the years 2002 to 2021, while ethnicity was imputed for the years 2003 to 2021 by the CDC. 6.2% of records had missing race or ethnicity, which were categorized as “other” in the analysis. It reported ethnicity as Hispanic and non-Hispanic and race as White, Black/African American, Asian, Native Hawaiian or other Pacific Islander, American Indian or Alaska Native, and more than one race. To increase statistical power for univariate and multivariate analyses, race and ethnicity were grouped into four categories: Hispanic, non-Hispanic White, non-Hispanic Black, and Other race (Native Hawaiian/Pacific Islander, American Indian/Alaska Native, more than one race, unknown, missing).

### Independent Variables

#### Demographics

The patient’s age was dichotomized into two groups: 2–12 months and 13–24 months based on their susceptibility to a UTI according to the American Academy of Pediatrics UTI guidelines [11]. The patient’s insurance was categorized as private, non-private, and other.

#### Visit Time and Location

To provide national and regional estimates, NHAMCS categorizes location into four regions: Northeast, Midwest, South, and West [8]. It also captures whether the visit took place in a metropolitan or non-metropolitan area [8]. To assess possible variations in urine testing that may occur during cold and flu seasons, the time of visit was categorized into Winter, Spring, Summer, and Fall.

#### Fever

Body temperature recorded during the visit was dichotomized into two categories: 38.0 °C – < 39.0 °C and ≥ 39.0 °C based on AAP UTI guideline [11, 12] categorization of fever as temperature ≥ 38.0 °C and increased UTI risk with temperature ≥ 39.0 °C. Circumcision status was not available.

### Outcome Variables

Our primary outcome was the frequency of urine testing, defined as urinalysis and/or urine culture at the time of visit based on the NHAMCS records. The urinalysis variable was available for the entire study period. However, the urine culture variable was available only for the years 2002 to 2004 and 2012 to 2021 (not collected for the years 2005 to 2011) [9]. We analyzed whether the frequency of urine testing in febrile children varies based on their race, ethnicity, and sex during the study period.

### Statistical Analysis

All analyses were performed with SAS (version 9.4) and accounted for the multistage probability sampling frame using survey commands. Descriptive analyses were used to present baseline characteristics of the study population. Categorical variables were reported as frequencies and survey-weighted percentages with 95% confidence intervals (CI). The presence of any urine testing was reported as a proportion with 95% CI. We grouped years into 2-year study periods to add power to our analysis, as recommended by the National Center for Health Statistics [8].

We assessed trends in the population-adjusted frequency of urine testing over time, stratified by race and ethnicity, using a linear test for trends across 2-year periods which are treated as continuous variables [13]. We performed univariate and multivariable logistic regression between predictor variables and the main outcome variable (use of any urine testing). Analyses were stratified by sex since AAP guidelines differ based on sex [4, 5] and the risk of UTI differs by sex. Variables (race and ethnicity) that were a priori felt to have a potential association with the frequencies of urine testing were included in our multivariate model. In addition, variables with a *p* < 0.20 in any of the univariate analyses (age, body temperature, season, and metropolitan statistical area; Supplementary Table 2) were also retained in our multivariable model to ensure no significant variables were overlooked; the cut-off of *p* < 0.20 in the univariate analysis was chosen based on precedent in literature [13, 14]. Estimates derived from the multivariable model included adjusted odds ratios (ORs) with 95% confidence intervals (CI).

To gain an understanding of contemporary urine testing frequencies, we performed a sub-analysis focused on data from the last 5 years (2017–2021) to analyze urine testing frequencies with respect to race and ethnicity stratified by sex. We then repeated the analyses outlined above with the 2017–2021 data.

## Results

### Study Population

**Fig. 1:**
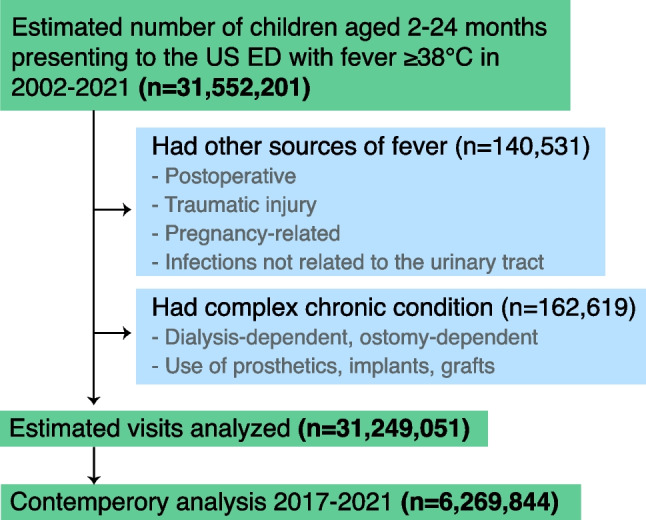
Derivation of our study population obtained from the National Hospital Ambulatory Medical Care Survey (NHAMCS) from 2002 to 2021. NHAMCS provides national estimates of total visits to the emergency departments (ED) in the United States (US) based on representative sampling of visits. Sub-analyses were conducted with the last 5 years of available data (2017–2021) to capture contemporary trends

Between 2002 and 2021, NHAMCS sampled 7268 emergency department visits by febrile children aged 2–24 months with recorded temperatures ≥ 38.0 °C (Fig. 1). Individuals with other sources of fever and complex chronic conditions were excluded. This nationally representative probability sampling was then estimated to be 31,249,051 total visits across the US (Table 1).

**Table 1:** Demographic characteristics of estimated us emergency department visits for febrile children aged 2–24 months. Data shown here are percentages with 95% confidence interval, unless otherwise indicated

Characteristics	All visits	Race and Ethnicity (2002–2021)			
		Hispanic	Non-Hispanic (NH)		
			NH-White	NH-Black	Other
Number of visits (*n*)	7,268	2,228	2,721	1,820	499
Estimated number of visits (*n*)	31,249,051	9,647,142	12,099,826	7,557,825	1,944,258
Percent of visits (%, 95% CI)	100.0	30.9 (27.9, 33.8)	38.7 (36.2, 41.3)	24.2 (21.4, 27.0)	6.2 (5.2, 7.3)
Age					
2–12 months	46.9 (45.2, 48.6)	46.3 (42.9, 49.7)	46.2 (43.3, 49)	47.7 (44.0, 51.3)	51.3 (45.6, 56.9)
13–24 months	53.1 (51.4, 54.8)	53.7 (50.3, 57.1)	53.8 (51.0, 56.7)	52.3 (48.7, 56.0)	48.7 (43.1, 54.4)
Sex					
Female	45.7 (44.1, 47.2)	45.8 (43.0, 48.6)	46.5 (44.1, 48.9)	44.4 (41.0, 47.8)	44.9 (38.7, 51.1)
Male	54.3 (52.8, 55.9)	54.2 (51.4, 57.0)	53.5 (51.1, 55.9)	55.6 (52.2, 59.0)	55.1 (48.9, 61.3)
Temperature (°C)					
38.0–38.9	53.3 (51.6, 55.0)	51.5 (48.4, 54.7)	53.2 (50.4, 56.0)	55.1 (52.0, 58.3)	55.0 (49.2, 60.9)
≥ 39.0	46.7 (45.0, 48.4)	48.5 (45.3, 51.6)	46.8 (44.0, 49.6)	44.9 (41.7, 48.0)	45.0 (39.1, 50.8)
Season					
Winter	30.5 (28.0, 32.9)	32.8 (28.3, 37.3)	29.5 (26.7, 32.2)	30.1 (26.4, 33.8)	55.0 (49.2, 60.9)
Spring	28.1 (25.5, 30.8)	28.0 (24.1, 31.9)	27.8 (25.1, 30.5)	27.1 (22.9, 31.3)	45.0 (39.1, 50.8)
Summer	21.3 (19.4, 23.3)	20.2 (17.2, 23.3)	22.2 (19.5, 24.8)	21.8 (18.6, 25.0)	55.0 (49.2, 60.9)
Fall	20.1 (18.0, 22.2)	19.0 (15.6, 22.3)	20.6 (18.1, 23.1)	21.0 (17.4, 24.7)	45.0 (39.1, 50.8)
Region					
Northeast	15.8 (13.3, 18.3)	19.6 (16.3, 22.9)	14.3 (11.2, 17.4)	12.8 (9.8, 15.8)	18.1 (14.3, 22.0)
Midwest	20.3 (16.7, 24.0)	12.6 (10.0, 15.2)	23.1 (18.4, 27.7)	24.7 (19.3, 30.1)	25.1 (19.0, 31.2)
South	44.3 (39.0, 49.5)	36.0 (29.7, 42.4)	45.7 (39.5, 51.9)	57.7 (50.6, 64.9)	23.7 (18.4, 29.0)
West	19.6 (16.0, 23.2)	31.8 (26.2, 37.4)	16.9 (13.2, 20.7)	4.7 (3.4, 6.1)	33.1 (26.0, 40.1)
Metropolitan statistical area					
Metropolitan	84.5 (80.3, 88.7)	90.4 (86.9, 93.9)	77.2 (70.6, 83.9)	86.9 (81.8, 91.9)	91.0 (86.3, 95.7)
Non-metropolitan	11.3 (7.4, 15.2)	4.3 (1.9, 6.6)	19.3 (12.8, 25.8)	8.7 (4.2, 13.1)	6.6 (2.1, 11.1)
Unknown	4.2 (2.9, 5.6)	5.4 (2.4, 8.3)	3.5 (2.3, 4.7)	4.5 (2.6, 6.4)	2.4 (0.9, 3.9)
Insurance					
Private	25.9 (23.9, 27.9)	18.0 (15.3, 20.6)	36.0 (32.8, 39.2)	17.7 (14.8, 20.6)	33.6 (27.4, 39.9)
Non-private	66.3 (63.9, 68.7)	74.7 (71.5, 77.8)	56.3 (52.8, 59.8)	74.6 (70.9, 78.3)	54.8 (48.6, 61.0)
Other/unknown	7.8 (6.1, 9.6)	7.3 (5.1, 9.6)	7.7 (5.4, 10.0)	7.7 (5.6, 9.8)	11.5 (7.1, 16.0)
Reason for health care visit					
Fever	80.3 (78.7, 81.8)	81.8 (79.7, 84.0)	79.5 (77.0, 81.9)	79.7 (76.9, 82.6)	79.6 (74.4, 84.8)
Urine testing					
Urinalysis and/or urine culture	20.0 (18.5, 21.6)	21.0 (18.2, 23.9)	20.3 (18.2, 22.5)	17.9 (15.1, 20.7)	21.7 (16.9, 26.5)

In our study population, nearly 8 out of 10 caretakers (80.3%, 95% CI 78.7, 81.8) listed fever as a primary reason for their child’s health care visit. The proportion of visits was higher in patients who were non-Hispanic White (38.7%, 95% CI 36.2, 41.3), male (54.3%, 95% CI 52.8, 55.9), over one year old (53.1%, 95% CI 51.4, 54.8), and presenting with mild fevers of 38–39.0 °C (53.3%, 95% CI 51.6, 55.0; Table 1). A disproportionately higher fraction of visits took place during the winter months (30.5%, 95% CI 28.0, 32.9), in the Southern regions (44.3%, 95% CI 39.0, 49.5), and in metropolitan areas (84.5%, 95% CI 80.3, 88.7; Table 1).

### Urine Testing Frequencies Over Two Decades

**Fig. 2:**
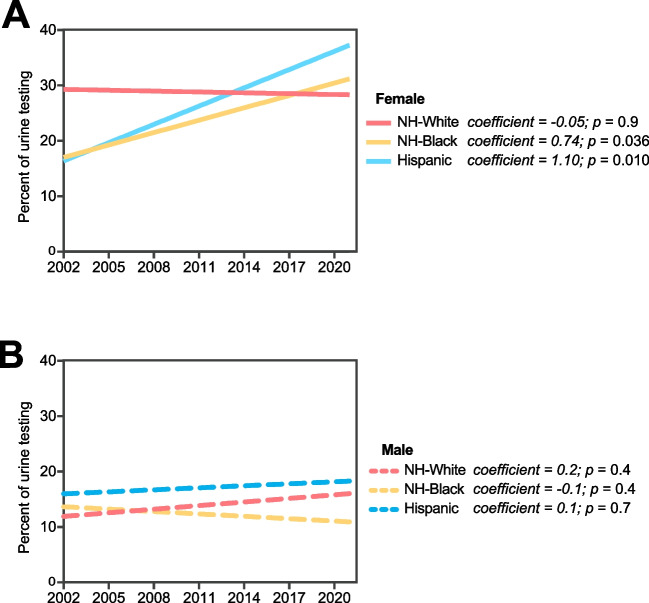
Urine testing trends among children aged 2–24 months from 2002 to 2021. **A**, **B:** Trendlines across time stratified by race, ethnicity, and sex. *p* values denote significance in trends of urine testing across time

In our study period, one in five children who presented to the ED with fever received urine testing (95% CI 18.5, 21.6; Table 1). Analysis of trends over time with respect to urine testing frequencies revealed significant differences when the data were stratified based on race, ethnicity, and sex (coefficients of 1.10 and 0.74 for Hispanic and non-Hispanic Black females, respectively; multivariate linear regression tests for trend, *p* < 0.05; Fig. 2A–B). The urine testing frequencies for non-Hispanic Black girls and Hispanic girls were lower compared to non-Hispanic White girls at the beginning of our study period, but the testing frequencies significantly increased over the following years, catching up to their White counterparts by the end of the study period (Fig. 2A–B).

### Contemporary Analysis of Urine Testing Frequencies (2017–2021)

**Fig. 3:**
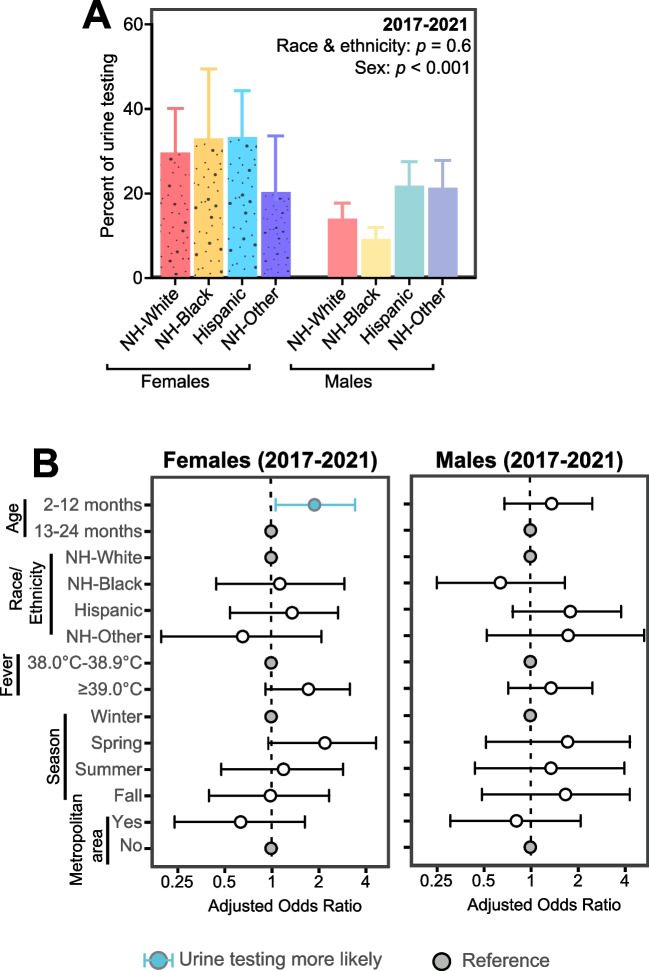
Contemporary analysis of urine testing among febrile children in the US Emergency Departments from 2017 to 2021. **A:** Urine testing percentages based on race, ethnicity, and sex. **B:** Logistic regression testing for factors associated with urine testing stratified by sex. Error bars represent 95% confidence interval

Given that trends of urine testing frequencies significantly changed over time and that our study period spanned across two decades, we conducted a sub-analysis using the last 5 years of available data (2017–2021) to capture contemporary trends in urine testing. Between 2017 and 2021, percent of urine testing was significantly higher in females compared to males in all race and ethnic groups (*p* < 0.001; Fig. 3A). Unlike our overall analysis, this 2017–2021 sub-analysis showed that race and ethnicity were no longer associated with urine testing frequencies (Hispanic females: 1.28, 95% CI 0.65, 2.53; Black females: 1.13, 95% CI 0.44, 2.89; Fig. 3B).

## Discussion

Our study uses nationally representative cross-sectional data from the National Hospital Ambulatory Medical Care Survey to show that non-Hispanic Black girls and Hispanic girls had lower frequencies of receiving urine tests compared to non-Hispanic White girls in early 2000s. However, urine testing for Hispanic females and non-Hispanic Black females significantly increased over time such that at the end of the observation period the degree of urine testing was similar across all race and ethnicity groups in females.

Our study showed that non-Hispanic Black girls and Hispanic girls with fever had lower urine testing frequencies compared to their non-Hispanic White counterparts at the beginning of the study period. One reason accounting for such discrepancy may be disparities in health care among Hispanic children presenting to the ED. Multiple studies have shown that compared to White children, Hispanic pediatric patients were 14% less likely to have their care needs classified as immediate/emergent [15], 3% less likely to be admitted to the hospital for management of UTIs or other causes [15, 16], and less likely to receive optimal treatment when admitted to the hospital for acute conditions such as subcutaneous abscesses among other conditions [17].

Race- and ethnicity-based differences in healthcare practices that we and others have reported may be exacerbated by clinical guidelines that use race and ethnicity as a proxy for genetic factors in algorithms for predicting the probability of disease. For example, the 2000 AAP guidelines stated White girls have a much higher rate (up to 15%) than Black girls of UTI, which may have resulted in lower urine testing rates for Black girls [18]. A recent study published in 2023 found that 126 pediatric clinical practice guidelines still used race in their decision-making [19]. Clinical guidelines are meant to standardize care, reduce provider implicit bias, and streamline the management of common diseases. However, incorporating race, which is a social construct, as a proxy for biological processes, is fundamentally flawed; it reinforces bias, discrimination, and vulnerabilities for people of color [5]. It ignores the historical context in which data have been gathered to justify biased practices that discriminate minority patients as was noted in our findings with lower odds of testing among Hispanic girls.

However, we observed a promising trend in increasing frequencies of testing among non-White female children, such that race and ethnicity were no longer significant factors in predicting urine testing in our sub-analysis using the last 5 years of available data (2017–2021). This encouraging trend may be a result of anti-racism efforts in medicine at every level from individual-level interventions to policy-level interventions. In 2020, the American Medical Association House of Delegates recognized racism as a serious threat to public health, to health equity, and a barrier to appropriate medical care [20]. Future studies will assess whether this trend will continue beyond the study period.

Important similarities and differences were observed when we compared our findings to a study that utilized a different data source from 26 US emergency departments in the Pediatric Health Information System in 2009–2019 that analyzed urine testing frequencies among febrile children [21]. In both of our studies, Hispanic female children had lower odds of receiving any urine testing compared to their White peers [21], indicating that it is a robust finding. Ramgopal et al. found significantly lower frequencies of urine testing in non-Hispanic Black children compared to non-Black children overall and after stratifying by sex [21]. Our study also found non-Hispanic Black girls to have lower frequencies of urine testing (Odds ratio 0.75, CI: 0.54–1.03), although it did not reach statistical significance at the 5% level (*p* = 0.07). While we observed a differential increase in urine testing frequencies for non-Hispanic Black and Hispanic female patients over time, Ramgopal et al. saw a decrease in urine testing after 2011 that affected females more than males [21]. Different data sources may account for such observed differences between the studies. NHAMCS is a nationally representative sample of ER visits from all participating US hospitals. Pediatric Health Information System collects data from pediatric-designated hospitals in the US, which means it does not capture care received by children in the hospitals that serve both children and adults [21–23]. Furthermore, overall urine testing frequencies were higher in Ramgopal et al. compared to our findings [21], which may reflect higher adherence to urine testing guidelines in pediatric-specific facilities. In fact, one study reported higher frequencies of pediatric bladder catheterization at the pediatric-specific emergency departments compared to general emergency departments [24].

Higher frequencies of urine testing were observed among younger (2–12 months), female children with higher fevers over ≥ 39.0 °C, all of which are important known risk factors for UTI in children according to the AAP UTI guidelines since 1999 [11, 25]. Studies estimated the overall prevalence of febrile UTIs in children less than 2 years old to be approximately 7%, with a higher prevalence of 8–8.4% in female children and a lower prevalence of 1.7–2% in uncircumcised male children [1, 26]. In fact, a calculator estimating the probability of UTI in febrile infants utilized the following clinical characteristics as risk factors: age less than 12 months, maximum temperature ≥ 39.0 °C, history of UTI, female or uncircumcised male, without other fever sources, and duration of fever ≥ 48 hours [27]. This validated calculator published in 2018 was found to reduce unnecessary testing, decrease missed UTIs, and reduce treatment delays [11, 27]. Our analysis indicates that the providers were using appropriate risk factors supported by evidence-based guidelines to decide when to administer urine tests.

Our study has limitations. One major limitation is that the results of the urine tests were not available, which limits our ability to assess the frequency of UTIs in our race, ethnicity, and sex groups. NHAMCS does not capture temperature measured at home at the onset of fever, fever duration, circumcision status, or history of UTIs, which are important risk factors that can influence urine testing frequencies. Although a few reports have questioned the reliability and accuracy of NHAMCS records [28], the CDC reassuringly reports that the error rate in the NHAMCS database is generally less than 2% [28]. NHAMCS captures race and ethnicity by various means including medical record extraction, which may be less accurate than direct patient reporting. Individualized data, such as zip codes for each emergency department, were not available, which prevented us from analyzing regional variation on a more granular level and from conducting sub-analyses to test differences in urine testing frequencies in low socioeconomic neighborhoods. Furthermore, we were not able to specifically identify settings that were pediatric-focused or the types and training levels of the treating providers, which may influence the degree of medical testing being conducted. Despite these limitations, our repeated cross-sectional study of a large, nationally representative, randomized sampling survey provided insights into a more generalizable reflection of the care children experienced on a national level during the observation period.

## Conclusion

Our study using a cross-sectional analysis of a nationally representative survey of febrile children presenting to the emergency departments revealed that Hispanic and non-Hispanic Black female patients had lower frequencies of urine testing compared to non-Hispanic White female patients. However, the trends of urine testing for Hispanic and non-Hispanic Black females increased over the study period, and the frequencies now match those of their White counterparts. Further work is necessary to understand whether these trends will impact health outcomes.

## Supplementary Information

Below is the link to the electronic supplementary material.

Supplementary file1 (XLSX 90 KB)

Supplementary file2 (XLSX 11 KB)

## Data Availability

Deidentified individual participant data will not be made available, since they were obtained from publicly available databases.

## Abbreviations

AAP: American Academy of Pediatrics
ED: Emergency department
CI: Confidence intervals
ICD 9: International Classification of Disease 9th revision codes
ICD 10: International Classification of Disease 10th revision codes
NHAMCS: National Hospital Ambulatory Medical Care Survey
OR: Odds ratios
US: United States
UTI: Urinary tract infection
